# Proposal of A New Bois Noir Epidemiological Pattern Related to ‘*Candidatus* Phytoplasma Solani’ Strains Characterized by A Possible Moderate Virulence in Tuscany

**DOI:** 10.3390/pathogens9040268

**Published:** 2020-04-07

**Authors:** Roberto Pierro, Alessandra Panattoni, Alessandro Passera, Alberto Materazzi, Andrea Luvisi, Augusto Loni, Marco Ginanni, Andrea Lucchi, Piero Attilio Bianco, Fabio Quaglino

**Affiliations:** 1 Department of Agriculture, Food and Environment (DAFE), University of Pisa, via del Borghetto 80, 56124 Pisa, Italy; rob.pierro@outlook.it (R.P.); alessandra.panattoni@unipi.it (A.P.); alberto.materazzi@unipi.it (A.M.); augusto.loni@unipi.it (A.L.); andrea.lucchi@unipi.it (A.L.); 2 Department of Agricultural and Environmental Sciences, Production, Landscape, Agroenergy (DiSAA), University of Milan, via Celoria 2, 20133 Milano, Italy; alessandro.passera@unimi.it (A.P.); fabio.quaglino@unimi.it (F.Q.); 3 Department of Biological and Environmental Sciences and Technologies, University of Salento, via Provinciale Monteroni, 73100 Lecce, Italy; andrea.luvisi@unisalento.it; 4 Agro-Environmental Research Center “Enrico Avanzi” (CiRAA), University of Pisa, via Vecchia di Marina 6, 56122 Pisa Italy; ginanni@tiscali.it; 5 Institute for Sustainable Plant Protection, National Research Council (IPSP-CNR), Strada delle Cacce 73, 10135 Turin, Italy

**Keywords:** grapevine yellows, *Reptalus quinquecostatus*, *Vitis vinifera* cv. Sangiovese, MLST, *stamp*, *secY*

## Abstract

Bois noir (BN), associated with ‘*Candidatus* Phytoplasma solani’ (CaPsol), is the most widespread disease of the grapevine yellows complex worldwide. In this work, BN epidemiology was investigated in a case study vineyard where an unusual CaPsol strain, previously detected only in other host plants, was found to be prevalent in grapevine. Experimental activities included: symptom observation; sampling of symptomatic vines, Auchenorrhyncha specimens, and weeds; molecular detection and typing of CaPsol strains; statistical analyses for determining possible relationships between CaPsol relative concentration, strain type, and symptom severity. Among insects, *Reptalus quinquecostatus* was the most abundant and was found to be highly infected by CaPsol, while *Hyalesthes obsoletus*, the main CaPsol vector, was not caught. Moreover, *R. quinquecostatus* harbored CaPsol strains carrying uniquely the *stamp* sequence variant St10, also identified as prevalent in vines and in the majority of weeds, and all the *secY* variants identified in the vineyard. Statistical analyses revealed that CaPsol strains carrying the St10 variant are not associated with severe symptoms, suggesting their possible moderate virulence. Based on such evidence, a new BN epidemiological pattern related to these CaPsol strains and involving grapevine, *R. quinquecostatus*, and/or weeds is proposed. Furthermore, the possible presence of other players (vectors and weeds) involved in CaPsol transmission to grapevines was highlighted.

## 1. Introduction

Grapevine yellows (GY) diseases, associated with phytoplasmas, constitute a major threat to viticulture worldwide. Bois noir (BN), one of the most important GY disease, is associated with ‘*Candidatus* Phytoplasma solani’ (CaPsol, taxonomic subgroup 16SrXII-A). CaPsol largely spread in Europe, Mediterranean regions and Iran, and has been sporadically reported from China, Chile, and South Africa [1,2,3]. In grapevine, BN induces symptoms undistinguishable from those of other GY diseases, such as desiccation of inflorescences, berry shrivel, leaf discolorations, reduction in growth, and irregular ripening of wood [4]. The cixiid *Hyalesthes obsoletus* Signoret, the main vector of BN [4], acquires CaPsol from its preferred host plants (*Convolvulus arvensis* L. and *Urtica dioica* L.), and occasionally transmits it to grapevine, a phytoplasma dead-end host [5,6]. Recently, typing of CaPsol strains using different molecular markers improved the knowledge of BN spreading, highlighting the crucial role of *Vitex agnus-castus* L. and *Crepis foetida* L. as the main CaPsol source plants for *H. obsoletus* [7,8], and proposing the involvement of other weeds and insects in BN epidemiology [9,10]. Moreover, combining field surveys, phytoplasma molecular characterization and transmission trials, *Reptalus panzeri* (Löw) was reported as a natural vector of CaPsol in Serbian vineyards [11], and another eight alternative vectors of CaPsol to grapevine (*Aphrodes makarovi* Zachvatkin, *Dicranotropis hamata* (Boheman), *Dictyophara europaea* (L.), *Euscelis incisus* (Kirschbaum), *Euscelidius variegatus* (Kirschbaum), *Laodelphax striatella* (Fallen), *Philaenus spumarius* (L.), and *Psammotettix alienus/confinis* (Dahlbom)) were identified in northern Italy [12]. 

Recently, a CaPsol strain, previously detected only in other host plants and characterized by carrying *stamp* gene sequence variant St10, was found prevalent in BN-affected vineyards in the Chianti Classico area, Tuscany. Due to its widespread distribution and moderate virulence, it was hypothesized that this CaPsol strain coevolved in the Tuscan vineyard agroecosystem, adapting to grapevine and other hosts [13,14]. Interestingly, previous studies conducted in vineyards located in the same area revealed the abundant presence of the cixiid *Reptalus quinquecostatus* (Dufour) [15], found able to transmit CaPsol to feeding medium and to periwinkle and suspected to be a CaPsol vector to grapevine [16,17], and of spontaneous weeds largely infected by CaPsol [18]. Such evidences opened a new intriguing scenario on the epidemiology of BN in Tuscany. 

In the present study, conducted in a Sangiovese vineyard located in the Chianti Classico area, a molecular epidemiology approach was applied to investigate such a scenario in order to identify the actors involved in CaPsol diffusion to grapevine, with a particular focus on the strain newly found in association with BN. Moreover, statistical analyses were carried out to find a possible correlation between symptom severity and CaPsol strains. 

## 2. Results

Partial results on CaPsol detection and *stamp* gene based typing in grapevines and *Reptalus quinquecostatus* (Dufour), included in this study, were preliminarily presented at IPWG Meeting 2019 (Valencia, Spain) [19]. To improve clarity and facilitate the readability and comprehension of the present study, such partial results were included in the manuscript.

### 2.1. Sampling and Phytoplasma Detection

The survey in the vineyard showed an incidence of GY symptoms equal to 6.7% (48 symptomatic vines out of 715). Based on symptom severity, 26 of these vines (54.2%) exhibiting severe symptoms were inserted in class 3; 12 of these vines (25%) exhibiting moderate symptoms were inserted in class 2; and 10 of these vines (20%) exhibiting mild symptoms were inserted in class 1 (Table 1). 

CaPsol was detected using real-time PCR in 45 out of 48 symptomatic vines [19] (Table 1). Phytoplasmas belonging to the 16SrI and 16SrV [including Flavescence dorée (FD) phytoplasma] taxonomic groups were not identified in any sample (grapevines, weeds, and insects). Real-time PCR analysis gave no amplification in negative controls (healthy grapevine control plant and reaction mixtures devoid of nucleic acids).

During the field surveys, asymptomatic weeds were collected and identified: leaf samples were collected from 54 weeds of nine species belonging to six families (Table 2). Real-time PCR analysis detected the presence of CaPsol in 33 out of 54 asymptomatic weeds. In detail, the species showing higher phytoplasma infection rate were *Convolvulus arvensis* L., *Pichris hieracioides* L., *Sonchus oleraceus* L., *Clematis vitalba* L., and *Potentilla reptans* L.; *Plantago major* L. was the sole species found not to be infected. *Ammi majus* L. and *Centaurium erythraea* Rafn. were found infected by CaPsol for the first time in the present study (Table 2).

By using sticky traps, 347 Auchenorrhyncha specimens of eight distinct taxonomic groups, four described at genus level and four at species level, were captured [19]. The prevalent taxonomic groups were *Reptalus quinquecostatus* (Dufour) (Rq) (186 specimens), *Phylaenus spumarius* (L.) (72 specimens), and *Zygina rhamni* Ferrari (57 specimens). The other insect taxonomic groups captured were: *Psammotettix* sp. (21 specimens), *Dictyophara europaea* (L.) (five specimens), *Neophilaenus* sp. (four specimens), *Cixius* sp. (one specimen), and *Macrosteles* sp. (one specimen). Real-time PCR analysis, conducted on the prevalent species Rq, detected the presence of CaPsol in 76 out of 186 Rq specimens [19]. CaPsol-infected samples (45 grapevines, 33 weeds, and 76 Rq) were utilized in molecular typing analyses.

### 2.2. ‘Candidatus Phytoplasma Solani’ Strain Typing

Nested PCR products of the genes *stamp* and *secY* were amplified from 143 (93%) and 95 (62%) out of 154 CaPsol-infected samples, respectively. Regarding the *stamp* gene, nested PCR products were obtained from 43 out of 45 vines, 67 out of 76 Rq specimens [19], and 33 out of 33 weeds (Table 1, Table 2 and Table 3). Regarding the *secY* gene, nested PCR products were obtained from 20 out of 45 vines, 15 out of 33 weeds, and 60 out of 76 Rq specimens (Table 1, Table 2 and Table 3). No amplification was obtained in negative controls.

**Figure 1:**
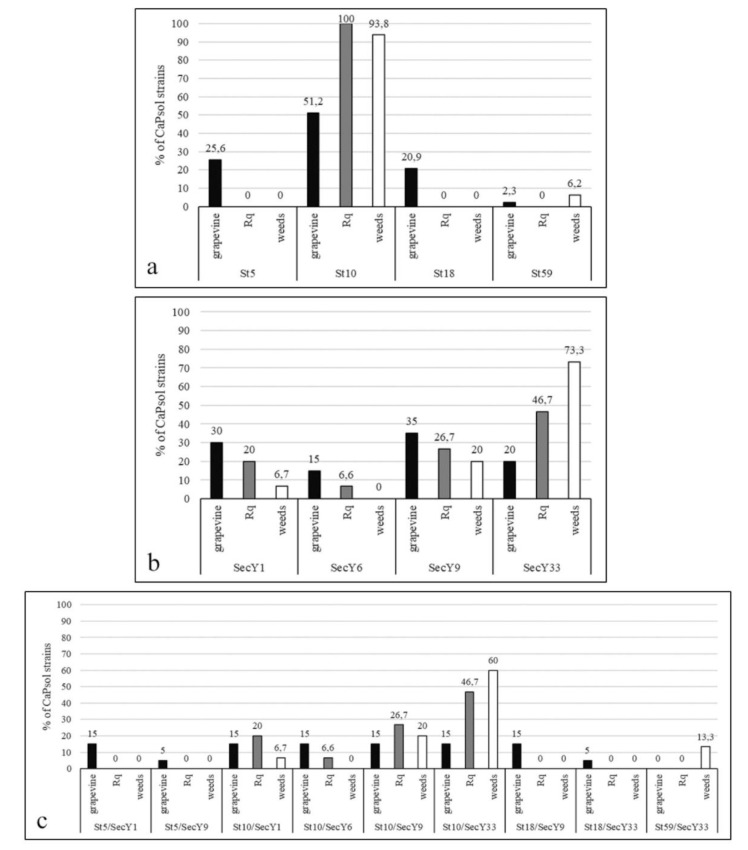
Prevalence (%) of *stamp* (**a**), *secY* (**b**) and *stamp/secY* (**c**) types of CaPsol strains identified in weeds, *R. quinquecostatus* specimens and grapevines in the examined vineyard.

Sequence identity analysis performed on 143 *stamp* gene amplicons allowed the identification of four sequence variants. Comparison with the *stamp* gene dataset, previously published in [13,14] and provided in Table S1, revealed that three of these sequence variants were identical to previously reported variants St5, St10, and St18; one variant, identified for the first time in this study and named St59, showed a similarity coefficient of 92.6% (35 SNPs) with St10. The nucleotide sequence of St59 was deposited in the NCBI GenBank repository under the Accession Number MN557212. The distribution of CaPsol *stamp* sequence variants in grapevines, weeds, and Rq samples was as follows: St10 was prevalent in all examined host plants and the only variant identified in Rq [19]; St5 and St18 were found exclusively in grapevine; lastly, St59 was found in one plant each of grapevine, *P. hieracioides*, and *C. arvensis* (Figure 1a).

Sequence identity analysis performed on 95 *secY* gene amplicons allowed the identification of four sequence variants. Comparison with the *secY* gene dataset, provided in Table S2, revealed that three of these sequence variants were identical to previously reported variants SecY1, SecY6, and SecY9; one variant, identified for the first time in this study and named SecY33, showed a similarity coefficient of 99.7% with SecY1 and SecY6 (2 SNPs vs. each). The nucleotide sequence of SecY33 was deposited in the NCBI GenBank repository under the Accession Number MN557211. The distribution of CaPsol *secY* sequence variants in grapevines, weeds, and Rq samples was as follows: SecY1, SecY9, and SecY33 were present in grapevines, weeds, and Rq, while SecY6 was only in grapevines and Rq. In detail, SecY1 and SecY9 were prevalent in grapevines, while SecY33 was prevalent in weeds and Rq (Figure 1b). Concerning the weeds, SecY33 was identified in all the species expect *A. majus*; SecY1 only in *P. hieracioides*; and SecY9 in *A. majus*, *P. hieracioides*, and *S. oleraceus* (Table 2).

The combination between *stamp* and *secY* sequence variants (St/SecY type) for each CaPsol strain and the comparison with the provided dataset (Table S3) allowed the identification of nine St/SecY types. The distribution of CaPsol St/SecY types in grapevines, weeds, and Rq samples was as follows: St10/SecY1, St10/SecY9, and St10/secY33 were present in grapevines, weeds, and Rq; St10/SecY6 in grapevine and Rq; St5/SecY1, St5/SecY9, St18/SecY9, and St18/SecY33 only in grapevine; and St59/SecY33 only in weeds, specifically *P. hieracioides* and *C. arvensis* (Table 1, Table 2 and Table 3). In particular, St10/SecY33 was the prevalent type in infected weeds and insects, and abundant in grapevine. It was not possible to determine a single prevalent St/SecY type in grapevine since six genetically distinct St/SecY types shared the same abundance (Figure 1c). 

### 2.3. Phylogenetic Analyses 

**Figure 2:**
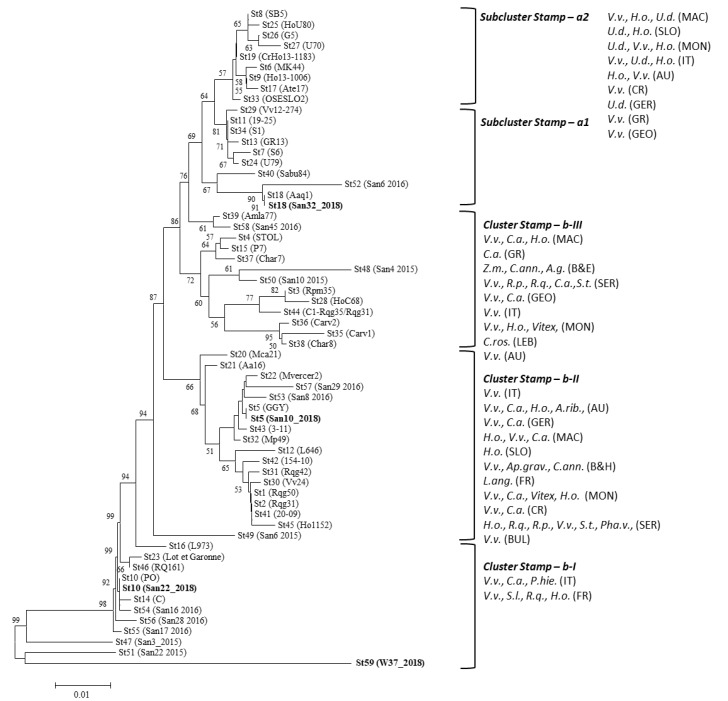
Unrooted phylogenetic tree inferred from *stamp* gene nucleotide sequences of CaPsol strains representative of *stamp* sequence variants previously described (Table S1) and identified in this study (Table 1, Table 2 and Table 3); minimum evolution analysis was performed using the neighbour-joining method and bootstrap replicated 1000 times. Names of strains are reported on the image and nucleotide sequences obtained in this study are in bold. Clusters are shown as delimitated by parentheses. Acronyms within clusters indicated phytoplasma hosts and origin. Hosts: *A.g., Apium graveolens; C.ann., Capsicum annum; C.a., Convolvulus arvensis; C.ros., Catharanthus roseus; H.o., Hyalesthes obsoletus; Pha.v., Phaseulus vulgaris; P.hie., Picris hieracioides; R.p., Reptalus panzeri; R.q., Reptalus quinquecostatus; S.l., Solanum lycopersicum; S.t., Solanum tuberosum; U.d., Urtica dioica; Vitex, Vitex agnus-castus; V.v., Vitis vinifera; Z.m., Zea mays*. Origin: AU, Austria; B and H, Bosnia and Herzegovina; BUL, Bulgaria; FR, France; GEO, Georgia; GER, Germany; IT, Italy; LEB, Lebanon; MAC, Republic of Macedonia; MON, Montenegro; SER, Serbia; SLO, Slovenia.

Four main clusters (nettle-related cluster a (divided in two subclusters a1 and a2), and bindweed-related clusters b-I, b-II, and b-III) are identified in the phylogenetic tree generated by the analysis of the *stamp* gene nucleotide sequences of the sequence variants identified in the present study and those reported in the dataset (Table S1). CaPsol strains were identified in the present study and characterized by the *stamp* sequence variants St5, St10, and the newly reported St59 grouped within the bindweed-related clusters b-I (St10 and St59) and b-II (St5); while the ones carrying the sequence variant St18 were positioned within the nettle-related subcluster a1 (Figure 2). 

**Figure 3:**
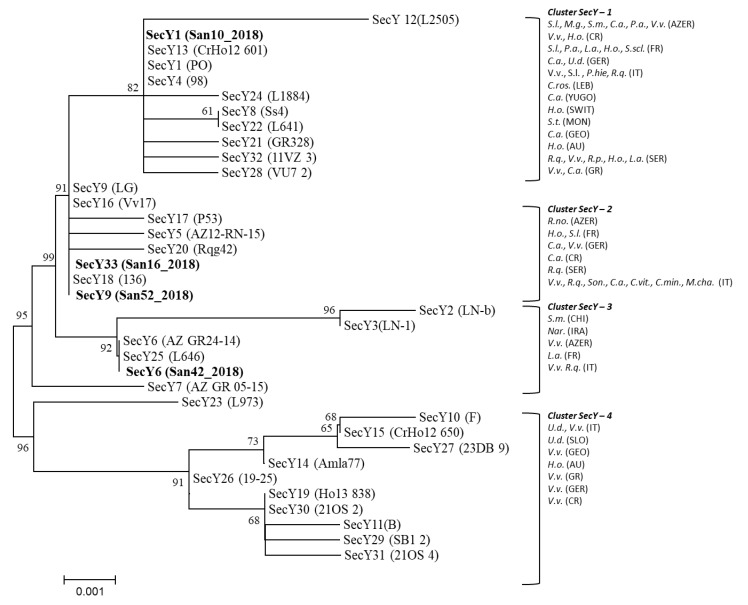
Unrooted phylogenetic tree inferred from *secY* gene nucleotide sequences of CaPsol strains representative of *secY* sequence variants previously described (Table S2) and identified in this study (Table 1, Table 2 and Table 3); minimum evolution analysis was performed using the neighbour-joining method and bootstrap replicated 1000 times. Names of strains are reported on the image and nucleotide sequences obtained in this study are in bold. Clusters are shown as delimitated by parentheses. Acronyms within clusters indicated phytoplasma hosts and origin. Hosts: *C.a., Convolvulus arvensis; C.min., Centaurea minus; C.ros., Catharanthus roseus; C.v., Clematis vitalba; H.o., Hyalesthes obsoletus; L.a., Lavandula angustifolia; M.cha., Matricaria chamomilla; Nar., Narcissus* sp.; *Pha.v., Phaseulus vulgaris; P.hie., Picris hieracioides; R.no., Reptalus noahi; R.p., Reptalus panzeri; R.q., Reptalus quinquecostatus; S.l., Solanum lycopersicum; S.m., Salvia miltiorrhiza; Son., Sonchus* sp.; *S.scl., Salvia sclarea; S.t., Solanum tuberosum; U.d., Urtica dioica; Vitex, Vitex agnus-castus; V.v., Vitis vinifera; Z.m., Zea mays*. Origin: AU, Austria; AZER, Azerbaijan; CH, China; CR, Croatia; FR, France; GEO, Georgia; GER, Germany; GR, Greece; IRA, Iran; IT, Italy; LEB, Lebanon; MON, Montenegro; SER, Serbia; SLO, Slovenia; SWIT, Switzerland; YUGO, Yugoslavia.

Four main clusters, here named *secY*-1 to -4, have been identified in the phylogenetic tree generated by the analysis of the *secY* gene nucleotide sequences of the sequence variants identified in the present study and those reported in the dataset (Table S2). CaPsol strains identified in the present study and characterized by the *secY* sequence variant SecY1 grouped within the cluster *secY*-1; those carrying the sequence variants SecY9 and SecY33 within the cluster *secY*-2; those characterized by the sequence variant SecY6 within the cluster *secY*-3 (Figure 3). 

**Figure 4:**
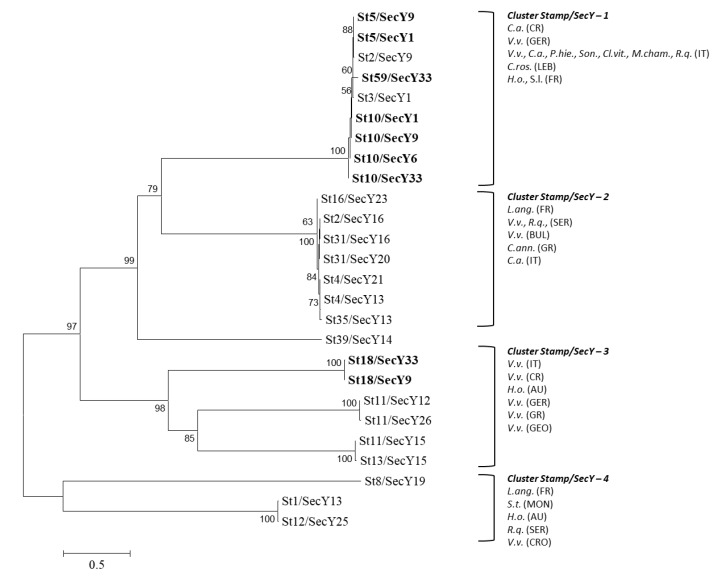
Unrooted phylogenetic tree inferred from *stamp*/*secY* gene nucleotide sequences of CaPsol strains representative of *stamp*/*secY* sequence variants previously described (Table S3) and identified in this study (Table 1, Table 2 and Table 3); minimum evolution analysis was performed using the neighbor-joining method and bootstrap replicated 1000 times. Names of strains are reported on the image and nucleotide sequences obtained in this study are in bold. Clusters are shown as delimitated by parentheses. Acronyms within clusters indicated phytoplasma hosts and origin. Hosts: *C.ann., Capsicum annum; C.a., Convolvulus arvensis; C.ros., Catharanthus roseus; C.v., Clematis vitalba; H.o., Hyalesthes obsoletus; L.a., Lavandula angustifolia; M.cha., Matricaria chamomilla; Nar., Narcissus* sp.; *Pha.v., Phaseulus vulgaris; P.hie., Picris hieracioides; R.q., Reptalus quinquecostatus; S.l., Solanum lycopersicum; S.m., Salvia miltiorrhiza; Son., Sonchus* sp.; *S.scl., Salvia sclarea; S.t., Solanum tuberosum; U.d., Urtica dioica; Vitex, Vitex agnus-castus; V.v., Vitis vinifera; Z.m., Zea mays*. Origin: AU, Austria; BUL, Bulgaria; CRO, Croatia; FR, France; GEO, Georgia; GER, Germany; GR, Greece; IT, Italy; LEB, Lebanon; MON, Montenegro; SER, Serbia.

Four main clusters, here named *stamp*/*secY*-1 to -4, have been identified in the phylogenetic tree generated by the analysis of the concatenated nucleotide sequences of *stamp* and *secY* genes of the St/SecY types identified in the present study and those reported in the dataset (Table S3). CaPsol strains identified in the present study and belonging to St/SecY types St5/SecY9, St5/SecY1, St59/SecY33, St10/SecY1, St10/SecY9, St10/SecY6, and St10/SecY33 grouped within the cluster *stamp*/*secY*-1; while those belonging to the types St18/SecY33 and St18/SecY9 within the cluster *stamp*/*secY*-3 (Figure 4). 

### 2.4. Relationship between Symptom Severity and ‘Candidatus Phytoplasma Solani’ Abundance or Strain

Statistically significant differences between symptom severity observed in symptomatic vines and ΔCq values (relative phytoplasma quantification) were obtained (*p* value = 0.000). In detail, phytoplasma ΔCq values were: 9.68 ± 1.72 in vines showing mild symptoms (class 1), 7.32 ± 2.22 in vines showing moderate symptoms (class 2), and 5.57 ± 1.98 in vines showing severe symptoms (class 3). 

On the contrary, no statistically significant differences were observed among ΔCq values obtained from symptomatic grapevines infected by CaPsol strains carrying distinct *stamp* sequence variants (*p* > 0.05).

No statistically significant differences were obtained in the distribution of CaPsol strains identified in the present study and carrying the *stamp* sequence variant St10 (common to grapevine, Rq, and weeds) in vines showing mild, moderate, or severe symptoms; while statistically significant differences were obtained in the distribution of CaPsol strains identified in the present study and carrying the *stamp* sequence variant St5, St18, and St59 (not common to all examined hosts), found prevalently in vines showing severe symptoms (Table 4). The same trend was confirmed by the statistical analysis of the distribution of the overall CaPsol strains, identified in the present (2017) and previous (2015, 2016) studies, in vines showing mild, moderate, or severe symptoms (Table 4). Considering the 2017 CaPsol strains carrying the four *stamp* sequence variants separately, a statistically significant difference was obtained only in the distribution of the strains carrying the nettle-related sequence variant St18, found prevalently in vines showing severe symptoms (Table 4).

## 3. Discussion

Survey on grapevine yellows (GY) symptoms showed that the disease incidence (6.7%) in the vineyard in Greve in Chianti was stable in comparison with the previous year (7.2%), and confirmed the prevalence of diseased vines exhibiting severe (class 3) symptoms (54.2% in 2017 vs. 47.2% in 2016) (13). Molecular detection of GY phytoplasmas confirmed the absence of flavescence dorée (FD) in the examined vineyard. In order to investigate Bois noir (BN) epidemiology and possible differences in the phytoplasma virulence, ‘*Candidatus* Phytoplasma solani’ (CaPsol) strains identified in grapevine, insects and weeds were characterized by sequence analyses of variable genes *stamp* and *secY*, largely utilized in previous studies [20,21,22], and comparison with sequence variant datasets by Pierro and colleagues (Tables S1–S3) [13,14]. 

As in previous studies on CaPsol multiple gene typing [13,14,21], nested-PCR reactions of *stamp* gene had the highest amplification rate in samples found to be CaPsol-infected by real-time PCR assays. Molecular typing showed the prevalence of CaPsol strains carrying the *stamp* gene sequence variants St5, St10, and St18 within the CaPsol population identified in diseased grapevines in 2017, as found in previous years 2015 and 2016 [13]. Thirteen CaPsol strains, each identified in 2015 and 2016 only in one symptomatic vine, were not detected in 2017. These strains, rarely found in grapevines, could have a minor role in BN epidemiology. 

The CaPsol strains carrying the *stamp* gene sequence variant St10, found to be prevalent in BN-affected vines in the examined vineyard in the years 2015, 2016 [13], and 2017 [19], this work was previously detected only in herbaceous host plants. Other studies conducted in vineyards located in the same area revealed the abundant presence of the cixiid *Reptalus quinquecostatus* (Dufour) (Rq), found able to transmit CaPsol to feeding medium and to periwinkle, and suspected to be a CaPsol vector to grapevine [15,16,17].

A survey on the Auchenorrhyncha highlighted the large prevalence of Rq within and at the borders of the studied vineyard, in which no specimens of the main vector *Hyalesthes obsoletus* Signoret were captured. Moreover, Rq specimens were found to be highly infected (40.8%) by CaPsol strains characterized uniquely by the *stamp* sequence variant St10, as preliminarily reported [19], and by *secY* sequence variants (SecY1, SecY6, SecY9, and SecY33) identified as also being prevalent in diseased vines and in the majority of the examined weed species. This evidence opens the intriguing hypothesis suggesting the existence of a new epidemiological pattern of CaPsol in Tuscany vineyards including grapevine, Rq, and weeds. However, transmission trials conducted in controlled conditions demonstrated the capability of Rq to transmit CaPsol to periwinkle but not to grapevine [17]. Due to the absence of the experimental proof demonstrating the Rq vectoring activity of CaPsol to grapevine, it was suggested that Rq could transmit CaPsol only to wild plant reservoirs (mainly bindweeds) present in the vineyard and its surroundings, increasing the inoculum sources for other known vectors (*H. obsoletus* or *Reptalus panzeri* (Löw)) able to acquire the phytoplasma from such plants and readily transmit it to grapevine [11,17]. In a previous study carried out in France, Rq was found to carry different CaPsol strains characterized by distinct *stamp* sequence variants [17]. Surprisingly, in the examined vineyard Rq specimens were found to harbor exclusively CaPsol strains carrying the *stamp* sequence variant St10. As the protein coded by *stamp* gene is an antigenic membrane protein, known to be involved in the mechanism determining the phytoplasma-vector specific recognition and interaction [23], it could be hypothesized that Rq population is extremely specialized for interacting with the CaPsol strains carrying the *stamp* sequence variant St10. Thus, transmission trials are necessary to verify the capability of Rq to transmit St10 CaPsol strains to grapevine.

Furthermore, the exclusive identification of CaPsol strains carrying the *stamp* sequence variants St5 and St18 in grapevine could suggest the presence of other vectors able to transmit to grapevine such CaPsol strains. It is interesting to note that three insect species, recently identified as alternative vectors of CaPsol to grapevine (*Phylaenus spumarius* (L.), *Psammotettix* spp., and *Dictyophara europaea* (L.)), were found during the survey on Auchenorrhyncha carried out in the vineyard in Greve in Chianti. In detail, transmission trials demonstrated that such insects are able to vector to grapevine CaPsol carrying the sequence variant St5 [12]. Moreover, based on the preliminary results obtained in this study about the weeds, the absence of St5 and St18 CaPsol strains in the examined weeds can suggest that other insect vectors could be able to acquire and transmit CaPsol using the infected vines as phytoplasma source. 

In this study, the relationship between relative CaPsol concentration (ΔCq values), CaPsol strain types, and different symptom severity observed in grapevines was investigated. No statistically significant differences were found among ΔCq values of genetically distinct CaPsol strains. On the other hand, statistical analysis showed that higher relative concentrations of the phytoplasma were associated with vines showing severe symptoms, and lower relative concentrations were associated with vines showing milder symptoms (*p* < 0.05). These data could suggest the existence of a positive relationship between symptom intensity in grapevine and the relative abundance of the phytoplasma. It could be also assumed that higher phytoplasma concentration in plant tissues implies more severe and widespread symptomatology in grapevine, at least for the cv. Sangiovese. Our results are in accordance with previous studies conducted on phytoplasma strains associated with FD [24,25,26]. 

Previous studies showed statistically significant differences in the distribution of CaPsol strains, carrying diverse *stamp* sequence variants, in grapevines with different symptom severity. Thus, it was hypothesized that these CaPsol strains can be characterized by a possible range of virulence [13,14]. In the present study, statistical analyses, conducted on CaPsol strains identified in the Greve in Chianti vineyard from 2015 to 2017 [133], this study revealed that CaPsol strains carrying *stamp* variants St5 and St18, identified in grapevine but not in Rq and in weeds, are associated prevalently with vines exhibiting severe symptoms, suggesting their possible high virulence. On the other hand, no statistically significant differences were found in the distribution of CaPsol strains carrying the *stamp* variant St10, identified in grapevine, Rq and weeds, in vines showing mild, moderate, and severe symptoms, suggesting their possible moderate virulence. This evidence reinforces the hypothesis that St10 CaPsol strains, largely prevalent in Tuscany and associated to a possible moderate virulence [13], can be co-evolved with and adapted to the host system grapevine—Rq—weeds in the vineyard agroecosystem in the Chianti Classico area.

In the last few years, numerous studies revealed that the BN epidemiology is more complex than previously postulated, reporting new relevant plant reservoirs (*Vitex agnus-castus* L. and *Crepis foetida* L.) for the known CaPsol vectors, and identifying some of the unknown alternative vectors [7,8,12]. Here, a new BN epidemiological pattern (grapevine—Rq—weeds) related to St10 CaPsol strains and milder symptoms is proposed. Moreover, the experimental evidence of the present study highlights the possible presence of other players (vectors and weeds) involved in CaPsol transmission to and diffusion among grapevines. 

## 4. Materials and Methods

### 4.1. GY Symptom Observation and Collection of Plant and Insect Samples

Field surveys were conducted in an organic Sangiovese vineyard (715 plants) located in Greve in Chianti (Chianti Classico area, Florence province), where, in a previous study, the CaPsol strain carrying the *stamp* gene sequence variant St10 was found to be prevalent in BN-affected grapevines (13). In September 2017, each GY symptomatic vine was visually assessed and attributed to a class of symptom severity, as previously reported [13] About 10 leaves were collected from each symptomatic vine. 

In July, weeds reported as potential CaPsol host plants [9,18] and weeds abundant in the vineyard were randomly collected at the inter-row areas and at the borders of the vineyard and identified in accordance with a dichotomous key.

In July, 10 yellow sticky traps were placed inside the vineyard and at its borders based on a regular grid ensuring the homogeneous coverage of the examined area. The traps, positioned on the wire supporting the grapevine canopy (inside the vineyard) and on driven poles at 0.5 m above the ground (at the borders), were substituted weekly [9] to monitor and capture potential insect vectors. Collected specimens were morphologically identified at genus/species level by stereomicroscope. Insects belonging to the species *Reptalus quinquecostatus* (Dufour) (Rq), previously found as an abundant putative vector in the examined area [15], were stored in ethanol 95% at 4 °C.

### 4.2. Total nucleic Acids Extraction and Phytoplasma Detection

Total nucleic acids (TNAs) were extracted from the leaf veins of vine and weed samples with CTAB-based buffer [13]. Insect TNAs were extracted from captured Rq specimens as previously described [27]. 

Specific detection of phytoplasmas associated with BN, Flavescence dorée (FD, taxonomic group 16SrV), and Aster Yellows (AY, taxonomic group 16SrI) was carried out by the amplification of 16S ribosomal DNA through TaqMan assay [28], using the Rotor-Gene Q (Qiagen, Germany). TNAs extracted from the leaf veins of a healthy Sangiovese grapevine plant, maintained in the greenhouse of the Department of Agriculture, Food and Environment (University of Pisa, Italy), and a reaction mixture devoid of TNAs were used as negative controls. To avoid contamination in the amplification reaction, no positive controls were utilized. The relative quantification of phytoplasmas in each sample was calculated using the formula: ΔCq = Cqp−Cqg, where ΔCq is the normalized value, Cqp is the Cq obtained from amplification of phytoplasmatic *16S rRNA* gene, and Cqg is the Cq obtained from amplification of grapevine chaperonin gene, the endogenous control used in the reaction [28,29].

### 4.3. Molecular Typing and Phylogeny of ‘Candidatus Phytoplasma Solani’ Strains

CaPsol strains detected in vines, weeds, and insects were typed by nucleotide sequence analyses of PCR-based amplicons of the non-ribosomal genomic regions *stamp* and *secY*. In detail, fragments of these genes were amplified in nested PCRs [30,31], carried out including the same controls described above for TaqMan assay, and sequenced (5X coverage per base position) by a commercial service (Eurofins Genomics, Germany). Nucleotide sequences of *stamp* and *secY* genes were aligned by ClustalW Multiple Alignment and analyzed by Sequence Identity Matrix within the software Bioedit v. 7.0.5.3 [32]. Unique *stamp* and *secY* nucleotide sequences, identified in this study, were attributed to sequence variants by their comparison with sequences previously deposited in GenBank and listed in reference datasets previously published [13,14] and provided in the present study (Tables S1–S3). No correspondence was present between the names of sequence variants listed in the reference datasets here utilized and those submitted in the GenBank database in another study [33]. For each *stamp* and *secY* sequence variant never reported before this study, one representative nucleotide sequence was deposited in the NCBI GenBank. Moreover, the collective type (*stamp*/*secY*) was determined by combining the sequence variant of both genes for each sample. A representative CaPsol strain for each *stamp*, *secY*, and *stamp*/*secY* type was included in phylogenetic analysis generating unrooted phylogenetic trees (Minimum Evolution method, Jukes-Cantor model, Bootstrap replication 1000), using MEGAX software [34].

### 4.4. Statistical Analysis

In a previous work, CaPsol strains, identified in 2015 and 2016 in the same vineyard examined in the present study and carrying distinct *stamp* gene sequence variants, were found to be differentially distributed in grapevines exhibiting mild, moderate, and severe symptoms. Based on this evidence, it was hypothesized that these CaPsol strains can be characterized by a possible range of virulence [13]. To confirm this hypothesis, the possible association between CaPsol strains carrying distinct *stamp* gene sequence variants, identified in this (2017) and in the previous study (2015, 2016) [13], and symptom severity classes observed in vines were evaluated using Chi square test. To determine if symptom severities were associated with different phytoplasma relative abundance in the vines, ΔCq values were compared between symptom severity classes and CaPsol strain types through one-way ANOVA, followed by Tukey HSD test, using SPSS statistical package for Windows, v. 24.0 (IBM Corporation, Armonk, NY) (*p* < 0.05).

## Figures and Tables

**Table 1:** Symptom severity class, relative abundance, and strain typing of CaPsol strains identified in grapevines located in the Greve in Chianti vineyard.

Vine Sample ID	Symptom Severity Class ^a^	CaPsol Relative Abundance and Typing ^b^		
		ΔCq ^c^	*stamp* Sequence Variant ^d^	*secY* Sequence Variant ^d^
G13	1	11.3	St10	SecY9
G14	1	10.3	St10	−
G27	1	10.4	St10	SecY33
G29	1	−	−	−
G37	1	8.36	St10	SecY6
G40	1	9.39	St10	−
G61	1	−	−	−
G70	1	7.45	St10	−
G72	1	7.89	St10	−
G74	1	12.4	St5	−
G4	2	9.03	St59	−
G7	2	5.56	St10	−
G24	2	8.35	St10	−
G34	2	7.59	St18	−
G45	2	10.3	−	−
G55	2	6.35	St5	SecY1
G59	2	3.66	St5	−
G60	2	8.62	St5	−
G62	2	3.62	St10	SecY6
G68	2	6.35	St10	
G71	2	9.51	St18	SecY33
G73	2	8.99	St10	SecY33
G1	3	3.43	St10	−
G2	3	8.17	St10	SecY1
G3	3	8.82	−	−
G6	3	4.21	St18	SecY9
G10	3	6.86	St5	SecY1
G12	3	12.3	St5	SecY1
G11	3	4.49	St18	−
G8	3	9.26	St5	−
G16	3	4.81	St10	SecY33
G18	3	6.17	St18	−
G21	3	3.65	St10	−
G22	3	4.25	St10	SecY9
G31	3	5.44	St5	−
G32	3	3.45	St18	SecY9
G33	3	5.91	St5	−
G38	3	4.63	St10	SecY1
G42	3	2.14	St10	SecY6
G43	3	3.07	St10	−
G44	3	3.87	St10	SecY1
G48	3	7.67	St18	−
G52	3	6.76	St5	SecY9
G54	3	−	−	−
G58	3	7.25	St18	−
G67	3	4.91	St10	SecY9
G69	3	6.08	St18	SecY9
G75	3	8.34	St5	−

^a^, symptom severity: mild (1); moderate (2); severe (3); ^b^, symbol "−": negative to amplification reaction; ^c^, ΔCq: normalized value of CaPsol relative quantification; ^d^, CaPsol gene sequence variant determined by nucleotide sequence identity value versus sequence variants of the published datasets [13,14].

**Table 2:** CaPsol identification and typing in collected weeds.

Family	Species	No. of Samples Infected/Collected	Sample ID	*stamp* Sequence Variant ^a,b^	*secY* Sequence Variant ^a,b^
Apiaceae	*Ammi majus* L.	2/5	W32	St10	−
			W55	St10	SecY9
Asteraceae	*Centaurium erythraea* Rafn.	1/2	W23	St10	SecY33
Asteraceae	*Matricaria chamomilla* L.	1/7	W31	St10	SecY33
Asteraceae	*Pichris hieracioides* L.	7/9	W39	St59	SecY33
			W40	St10	−
			W43	St10	−
			W44	St10	SecY33
			W50	St10	−
			W51	St10	SecY1
			W65	St10	SecY9
Asteraceae	*Sonchus oleraceus* L.	6/8	W30	St10	−
			W52	St10	SecY33
			W53	St10	−
			W56	St10	SecY33
			W57	St10	−
			W61	St10	SecY9
Convolvulaceae	*Convolvulus arvensis* L.	12/17	W21	St10	SecY33
			W26	St10	SecY33
			W28	St10	−
			W37	St59	SecY33
			W38	St10	−
			W41	St10	−
			W42	St10	−
			W45	St10	−
			W48	St10	−
			W58	St10	−
			W59	St10	SecY33
			W60	St10	−
Plantaginaceae	*Plantago major* L.	0/2			
Ranunculaceae	*Clematis vitalba* L.	2/2	W35	St10	−
			W36	St10	SecY33
Rosaceae	*Potentilla reptans* L.	2/2	W24	St10	−
			W25	St10	−

^a^, symbol “−”: negative to amplification reaction; ^b^,CaPsol gene sequence variant determined by nucleotide sequence identity value versus sequence variants of the published datasets [13,14].

**Table 3:** Typing of CaPsol strains identified in *R. quinquecostatus* specimens captured inside and at the border of the Greve in Chianti vineyard.

No. of CaPsol Strains in Rq Specimens	*stamp* Sequence Variant ^b^	*secY* Sequence Variant ^a,b^
12	St10	SecY1
4	St10	SecY6
16	St10	SecY9
28	St10	SecY33
7	St10	−

^a^, symbol “−”: negative to amplification reaction; ^b^,CaPsol gene sequence variant determined by nucleotide sequence identity value versus sequence variants of the published datasets [13,14].

**Table 4:** Distribution of CaPsol strains carrying common and uncommon *stamp* sequence variants in vines exhibiting mild, moderate, and severe symptoms.

*stamp* Sequence Variant	Year	No. of CaPsol Strains in Vines with Different Symptom Severity			Goodness of Fit	
		Class 1	Class 2	Class 3	Chi Square	P Value
common (St10)	2017	7	5	10	2.411	0.299
	2015-17	12	11	21	3.751	0.153
uncommon (St5, St18, St59)	2017	1	6	14	12.286	0.002
	2015-17	10	21	34	12.353	0.002

## Supplementary Materials

The following are available online at https://www.mdpi.com/2076-0817/9/4/268/s1, Table S1: Sequence variants dataset of the gene *stamp* among ’*Ca*. P. solani’ strains available in GenBank, Table S2: Sequence variants dataset of the gene *secY* among ’*Ca*. P. solani’ strains available in GenBank, Table S3: Sequence variants dataset of the *stamp/secY-*types among ’*Ca*. P. solani’ strains available in GenBank.
