# Supplementary material for: Proposal of A New Bois Noir Epidemiological Pattern Related to ‘Candidatus Phytoplasma Solani’ Strains Characterized by A Possible Moderate Virulence in Tuscany
Source: Pathogens. 2020 Apr 7;9(4):268. doi: 10.3390/pathogens9040268 (PMC7238118; doi:10.3390/pathogens9040268)
Supplement: Supplementary file 1 [file pathogens-09-00268-s001.pdf]

Supporting Material

Table S1. Sequence variants Dataset of the gene *stamp* among '*Ca*. P. solani' strains available in GenBank.

Sequence Variant	Strain	Host	Location	Acc. N. *stamp*
St1	Rqg50	*Reptalus quinquecostatus*	Serbia	KC703019
St1	11-Nov	*Vitis vinifera*	Bosnia Herzegovina	KP739852
St1	115/11	*Vitis vinifera*	Italy	KJ145337
St1	17-Nov	*Vitis vinifera*	Bosnia Herzegovina	KP739854
St1	20MN	*Vitis vinifera*	Montenegro	KJ926068
St1	353/11	*Vitis vinifera*	Italy	KJ145338
St1	45MN	*Convolvulus arvensis*	Montenegro	KJ926069
St1	72MN	*Vitex agnus-castus*	Montenegro	KJ926070
St1	Ag4a	*Vitis vinifera*	Italy	KJ145377
St1	B1	*Vitis vinifera*	Italy	KJ145378
St1	C45	*Convolvulus arvensis*	Macedonia	KP337319
St1	CrAr12_722_2	*Anaceratagallia ribauti*	Austria	KJ469722
St1	CrHo12_721	*Hyalesthes obsoletus*	Austria	KJ469722
St1	G21-13	*Vitis vinifera*	Bosnia Herzegovina	KP739856
St1	G22-13	*Vitis vinifera*	Bosnia Herzegovina	KP739849
St1	G23-13	*Vitis vinifera*	Bosnia Herzegovina	KP739846
St1	G24-13	*Vitis vinifera*	Bosnia Herzegovina	KP739847
St1	G4-13	*Vitis vinifera*	Bosnia Herzegovina	KP739853
St1	G6-13	*Vitis vinifera*	Bosnia Herzegovina	KP739848
St1	Gb1	*Phaseulus vulgaris*	Serbia	KM977907
St1	Ho375	*Hyalesthes obsoletus*	Montenegro	KJ926071
St1	Ho66-2	*Hyalesthes obsoletus*	Montenegro	KJ926072
St1	HoC202	*Hyalesthes obsoletus*	Macedonia	KP337320
St1	Mp46	*Vitis vinifera*	Italy	KJ145379
St1	P25/11	*Vitis vinifera*	Italy	KJ145339
St1	PM1	*Solanum tuberosum*	Montenegro	KU588188
St1	PM2	*Solanum tuberosum*	Montenegro	KU588189
St1	PS8	*Solanum tuberosum*	Serbia	KP877599
St1	PS8Ho	*Hyalesthes obsoletus*	Serbia	KP877600
St1	PS8Rp	*Reptalus panzeri*	Serbia	KP877601
St1	PS9	*Solanum tuberosum*	Serbia	KP877602
St1	Rpg47	*Reptalus panzeri*	Serbia	KC703020
St1	Vv12_Ill6	*Vitis vinifera*	Austria	KJ469722
St1	Vv5	*Vitis vinifera*	Serbia	KC703021
St2	Rqg31	*Reptalus quinquecostatus*	Serbia	KC703017
St2	Br8	*Convolvulus arvensis*	Croatia	KJ573597
St2	C2_Rgg50	*Apium graveolens*	Bosnia Herzegovina	KU295506
St2	Ho41-2	*Hyalesthes obsoletus*	Montenegro	KJ926065
St2	P10	*Capsicum annuum*	Bosnia Herzegovina	KU295504
St2	P6	*Capsicum annuum*	Bosnia Herzegovina	KU295502
St2	PS4	*Solanum tuberosum*	Serbia	KP877588

Sequence Variant	Strain	Host	Location	Acc. N. *stamp*
St2	PS4Ho	*Hyalesthes obsoletus*	Serbia	KP877589
St2	PS5	*Solanum tuberosum*	Serbia	KP877590
St2	PS5Ho	*Hyalesthes obsoletus*	Serbia	KP877591
St2	PS5Rp	*Reptalus panzeri*	Serbia	KP877592
St2	PS6	*Solanum tuberosum*	Serbia	KP877593
St2	PS6Ho	*Hyalesthes obsoletus*	Serbia	KP877594
St2	PS6Rq	*Reptalus quinquecostatus*	Serbia	KP877595
St2	Vv12_Kn6	*Vitis vinifera*	Austria	KJ469724
St2	Vv17	*Vitis vinifera*	Serbia	KC703018
St3	16MN	*Vitis vinifera*	Montenegro	KJ926073
St3	30-Sep	*Vitis vinifera*	Bosnia Herzegovina	KP739851
St3	43MN	*Convolvulus arvensis*	Montenegro	KJ926074
St3	79MN	*Vitex agnus-castus*	Montenegro	KJ926075
St3	Ho389	*Hyalesthes obsoletus*	Montenegro	KJ926076
St3	MK66	*Vitis vinifera*	Macedonia	KF957608
St3	P5	*Capsicum annuum*	Bosnia Herzegovina	KU295501
St3	P7	*Catharanthus roseus*	Lebanon	FN813258
St3	PS7	*Solanum tuberosum*	Serbia	KP877596
St3	PS7Ho	*Hyalesthes obsoletus*	Serbia	KP877597
St3	PS7Rp	*Reptalus panzeri*	Serbia	KP877598
St3	Rpm35	*Reptalus panzeri*	Serbia	KC703015
St3	Vv12_751	*Vitis vinifera*	Austria	KJ469723
St4	G2	*Vitis vinifera*	Macedonia	KP337318
St4	GR328	*Capsicum annuum*	Greece	FN813253
St4	Ho10-2	*Hyalesthes obsoletus*	Montenegro	KJ926067
St4	MB11	*Zea mays*	Bosnia Herzegovina	KU295509
St4	MB4	*Zea mays*	Bosnia Herzegovina	KU295507
St4	MB6	*Zea mays*	Bosnia Herzegovina	KU295508
St4	PS1	*Solanum tuberosum*	Serbia	KP877583
St4	PS1Rp	*Reptalus panzeri*	Serbia	KP877584
St4	PS1Rq	*Reptalus quinquecostatus*	Serbia	KP877585
St4	Rpg39	*Reptalus panzeri*	Serbia	KC703009
St4	Rpm34	*Reptalus panzeri*	Serbia	KC703010
St4	Rqg60	*Reptalus quinquecostatus*	Serbia	KC703011
St4	STOL	*Capsicum annuum*	Serbia	FN813261
St4	Vexp Rpg11	*Reptalus panzeri*	Serbia	KC703013
St4	Vexp Rpm5	*Reptalus panzeri*	Serbia	KC703014
St4	Vv21	*Vitis vinifera*	Serbia	KC703012
St5	215/11	*Vitis vinifera*	Italy	KJ145329
St5	287/11	*Vitis vinifera*	Italy	KJ145332
St5	315/11	*Vitis vinifera*	Italy	KJ145330
St5	425/11	*Vitis vinifera*	Italy	KJ145335
St5	78/11	*Vitis vinifera*	Italy	KJ145334
St5	Ca13_RF	*Convolvulus arvensis*	Austria	KJ469721

Sequence Variant	Strain	Host	Location	Acc. N. *stamp*
St5	CrHo12_601	*Hyalesthes obsoletus*	Austria	KJ469721
St5	GGY	*Vitis vinifera*	Germany	FN813256
St5	HoC205	*Hyalesthes obsoletus*	Macedonia	KP337315
St5	LA6_I_C	*Convolvulus arvensis*	Germany	JQ977720
St5	NGA9	*Hyalesthes obsoletus*	Slovenia	FN813262
St5	P136/11	*Vitis vinifera*	Italy	KJ145336
St5	P51/11	*Vitis vinifera*	Italy	KJ145331
St5	P75/11	*Vitis vinifera*	Italy	KJ145333
St5	Vv12_752	*Vitis vinifera*	Austria	KJ469721
St5	Vv12_754	*Vitis vinifera*	Austria	KJ469721
St5	San23_2015	*Vitis vinifera*	Italy	MF182869
St6	MK44	*Vitis vinifera*	Macedonia	KF957607
St6	S7	*Urtica dioica*	Slovenia	JQ977719
St7	S6	*Urtica dioica*	Italy	JQ977718
St8	49MN	*Urtica dioica*	Montenegro	KJ926078
St8	4MN	*Vitis vinifera*	Montenegro	KJ926077
St8	BN-Yan1	*Vitis vinifera*	Italy	KX151182
St8	Ho13_838	*Hyalesthes obsoletus*	Austria	KJ469720
St8	Ho13-8	*Hyalesthes obsoletus*	Montenegro	KJ926079
St8	HoU190	*Hyalesthes obsoletus*	Macedonia	KP337321
St8	S5	*Urtica dioica*	Italy	JQ977717
St8	SB5	*Vitis vinifera*	Croatia	FN813266
St9	60/11	*Vitis vinifera*	Italy	KJ145345
St9	7-Nov	*Vitis vinifera*	Croatia	KP274915
St9	Aa25	*Vitis vinifera*	Italy	KJ145387
St9	Aaq29	*Vitis vinifera*	Italy	KJ145388
St9	Ho13_1006	*Hyalesthes obsoletus*	Austria	KJ469718
St9	Mcil	*Vitis vinifera*	Italy	KJ145385
St9	S2	*Urtica dioica*	Slovenia	JQ977714
St10	LG	*Solanum lycopersicum*	France	FN813257
St10	PO	*Hyalesthes obsoletus*	France	FN813270
St10	San21_2015	*Vitis vinifera*	Italy	MF182868
St11	19-25	*Vitis vinifera*	Germany	FN813267
St11	33MN	*Vitis vinifera*	Montenegro	KJ926080
St11	67MN	*Urtica dioica*	Montenegro	KJ926081
St11	CrHo12_650	*Hyalesthes obsoletus*	Austria	KJ469716
St11	E	*Hyalesthes obsoletus*	Germany	FN813263
St11	G1	*Vitis vinifera*	Macedonia	KP337322
St11	GBr2	*Vitis vinifera*	Croatia	KJ573590
St11	GBr4	*Vitis vinifera*	Croatia	KJ573591
St11	GVu1	*Vitis vinifera*	Croatia	KJ573592
St11	GVu2	*Vitis vinifera*	Croatia	KJ573593
St11	H17	*Hyalesthes obsoletus*	Croatia	KJ573594
St11	H18	*Hyalesthes obsoletus*	Croatia	KJ573595

Sequence Variant	Strain	Host	Location	Acc. N. *stamp*
St11	H21	*Hyalesthes obsoletus*	Croatia	KJ573596
St11	Ho36-8	*Hyalesthes obsoletus*	Montenegro	KJ926082
St11	HoU17	*Hyalesthes obsoletus*	Macedonia	KP337323
St11	MK94	*Vitis vinifera*	Macedonia	KF957609
St12	L646	*Lavandula angustifolia*	France	FN813265
St13	GR13	*Vitis vinifera*	Greece	FN813264
St14	C	*Solanum lycopersicum*	France	FN813260
St15	P7	*Capsicum annuum*	Bosnia Herzegovina	KU295503
St15	Tsol89	*Vitis vinifera*	Georgia	KT184885
St15	Kiqu84	*Vitis vinifera*	Georgia	KT184885
St16	H299	*Hyalesthes obsoletus*	France	FN813254
St16	L973	*Lavandula angustifolia*	France	FN813255
St17	Ate17	*Vitis vinifera*	Italy	KJ145386
St18	266/11	*Vitis vinifera*	Italy	KJ145344
St18	Aaq1	*Vitis vinifera*	Italy	KJ145383
St18	Mdxsain	*Vitis vinifera*	Italy	KJ145384
St18	San2_2015	*Vitis vinifera*	Italy	MF182870
St19	CrHo13_1183	*Hyalesthes obsoletus*	Austria	KJ469719
St19	S3	*Urtica dioica*	Slovenia	JQ977715
St20	136/11	*Vitis vinifera*	Italy	KJ145340
St20	166/11	*Vitis vinifera*	Italy	KJ145343
St20	Ate7	*Vitis vinifera*	Italy	KJ145381
St20	Mca21	*Vitis vinifera*	Italy	KJ145382
St20	P10/11	*Vitis vinifera*	Italy	KJ145342
St20	P42/11	*Vitis vinifera*	Italy	KJ145341
St21	Aa16	*Vitis vinifera*	Italy	KJ145380
St22	Mvercer2	*Vitis vinifera*	Italy	KJ145375
St22	San24_2015	*Vitis vinifera*	Italy	MF182871
St23	Lot et Garonne	*Solanum lycopersicum*	France	FN813257
St24	HoU93	*Hyalesthes obsoletus*	Macedonia	KP337314
St24	U79	*Hyalesthes obsoletus*	Macedonia	KP337313
St25	HoU80	*Hyalesthes obsoletus*	Macedonia	KP337309
St26	G5	*Hyalesthes obsoletus*	Macedonia	KP337310
St26	HoU85	*Hyalesthes obsoletus*	Macedonia	KP337311
St27	U70	*Urtica dioica*	Macedonia	KP337312
St28	HoC68	*Hyalesthes obsoletus*	Macedonia	KP337316
St28	PS3	*Solanum tuberosum*	Serbia	KP877587
St29	Vv12_274	*Vitis vinifera*	Austria	KJ469717
St30	10MN	*Vitis vinifera*	Montenegro	KJ926066
St30	4-Sep	*Vitis vinifera*	Croatia	KP274914
St30	G25	*Vitis vinifera*	Macedonia	KP337317
St30	PS10Ho	*Hyalesthes obsoletus*	Serbia	KP877603
St30	PS10Rq	*Reptalus quinquecostatus*	Serbia	KP877604
St30	Vv24	*Vitis vinifera*	Serbia	KC703022

Sequence Variant	Strain	Host	Location	Acc. N. *stamp*
St31	BG4560	*Vitis vinifera*	Bulgaria	FN813252
St31	PS2	*Solanum tuberosum*	Serbia	KP877586
St31	Rqg42	*Reptalus quinquecostatus*	Serbia	KC703016
St32	Mp49	*Vitis vinifera*	Italy	KJ145376
St33	OSESLO2	*Hyalesthes obsoletus*	Slovenia	FN813269
St33	Rome15	*Hyalesthes obsoletus*	Italy	FN813268
St33	S4	*Urtica dioica*	Italy	JQ977716
St34	S1	*Urtica dioica*	Germany	JQ977713
St35	Carv1	*Convolvulus arvensis*	Georgia	KT184879
St36	Carv2	*Convolvulus arvensis*	Georgia	KT184880
St37	Char7	*Convolvulus arvensis*	Georgia	KT184881
St37	Kisi38	*Vitis vinifera*	Georgia	KT184881
St37	Rkat47	*Vitis vinifera*	Georgia	KT184881
St37	Sape51	*Vitis vinifera*	Georgia	KT184881
St37	Sape62	*Vitis vinifera*	Georgia	KT184881
St38	Char8	*Convolvulus arvensis*	Georgia	KT184882
St38	Sape19	*Vitis vinifera*	Georgia	KT184882
St38	GoMt25	*Vitis vinifera*	Georgia	KT184882
St39	Amla77	*Vitis vinifera*	Georgia	KT184883
St40	Sabu84	*Vitis vinifera*	Georgia	KT184884
St41	20-Sep	*Vitis vinifera*	Bosnia Herzegovina	KT766177
St42	154-10	*Vitis vinifera*	Bosnia Herzegovina	KP739855
St43	3-Nov	*Vitis vinifera*	Bosnia Herzegovina	KP739850
St44	C1_Rgg35/Rqg31	*Apium graveolens*	Bosnia Herzegovina	KU295505
St45	Ho1152	*Hyalesthes obsoletus*	Montenegro	KM977906
St46	RQ161	*Reptalus quinquecostatus*	France	LN823951
St47	San3_2015	*Vitis vinifera*	Italy	MF182872
St48	San4_2015	*Vitis vinifera*	Italy	MF182873
St49	San6_2015	*Vitis vinifera*	Italy	MF182874
St50	San10_2015	*Vitis vinifera*	Italy	MF182875
St51	San22_2015	*Vitis vinifera*	Italy	MF182876
St52	San6_2016	*Vitis vinifera*	Italy	MF182877
St53	San8_2016	*Vitis vinifera*	Italy	MF182878
St54	San16_2016	*Vitis vinifera*	Italy	MF182879
St55	San17_2016	*Vitis vinifera*	Italy	MF182880
St56	San28_2016	*Vitis vinifera*	Italy	MF182881
St57	San29_2016	*Vitis vinifera*	Italy	MF182882
St58	San45_2016	*Vitis vinifera*	Italy	MF182883
St59	W37_2018	*Convolvulus arvensis*	Italy	MN557212
St59	W39_2018	*Picris hieracioides*	Italy	MN557212

Table S2. Sequence variants Dataset of the gene *secY* among '*Ca*. P. solani' strains available in GenBank.

Sequence Variant	Strain	Host	Location	Accession Number
SecY1	AZ-TO41-08	*Solanum lycopersicum*	Azerbaijan	LT899857.1
SecY1	AZ-NEF40-08	*Mespilus germanica*	Azerbaijan	LT899856.1
SecY1	AZ-AU3-07	*Solanum melanogena*	Azerbaijan	LT899855.1
SecY1	AZ-PV1-07	*Capsicum annuum*	Azerbaijan	LT899854.1
SecY1	AZ3_CE3	*Prunus avium*	Azerbaijan	LT899853.1
SecY1	AZ_GR-21-14	*Vitis vinifera*	Azerbaijan	LT899842.1
SecY1	11VZ_4	*Vitis vinifera*	Croatia	HQ413160.1
SecY1	Ss24	*Salvia sclarea*	France	LT841330.1
SecY1	StolC	*Solanum lycopersicum*	France	AM992083.1
SecY1	Moliere	*Prunus avium*	France	AM992090.1
SecY1	L1088	*Lavandula*	France	HF969329.1
SecY1	PO	*Hyalestes obsoletus*	France	AM992082.1
SecY1	D	*Convolvulus arvensis*	Germany	JQ977710.1
SecY1	CH1	*Vitis vinifera*	Italy	AM992089.1
SecY1	T2_92	*Solanum lycopersicum*	Italy	AM992088.1
SecY1	P7	*Catharanthus roseus*	Lebanon	AM992091.1
SecY1	W51	*Picris hieracioides*	Italy	MT071089
SecY1	F30	*Reptalus quinquecostatus*	Italy	MT071090
SecY1	Red-Pepper	*Capsicum annuum*	Yugoslavia	AM992086.1
SecY2	LN-b	*Salvia miltiorrhiza*	China	KU600104.1
SecY3	LN-1	*Salvia miltiorrhiza*	China	KU600094.1
SecY3	SH-1	*Narcissus sp*	Iran	KY315180.1
SecY4	98	*Hyalestes obsoletus*	Switzerland	KT310178.1
SecY5	AZ13-H05	*Hyalestes obsoletus*	Azerbaijan	LT899847.1
SecY5	AZ12-RN-15	*Reptalus noahi*	Azerbaijan	LT899846.1
SecY6	San42_2018	*Vitis vinifera*	Italy	MT071091
SecY6	F32	*Reptalus quinquecostatus*	Italy	MT071092
SecY6	AZ_GR24-14	*Vitis vinifera*	Azerbaijan	LT899845.1
SecY7	AZ_GR_05-15	*Vitis vinifera*	Azerbaijan	LT899838.1
SecY8	Ss4	*Salvia sclarea*	France	LT841331.1
SecY9	HO-Ss41	*Hyalesthes obsoletus*	France	LT841329.1
SecY9	C	*Convolvulus arvensis*	Germany	JQ977709.1
SecY9	GGY	*Vitis vinifera*	Germany	AM992093.1
SecY9	LG	*Lycopersicum esculentum*	France	AM992092.1
SecY9	Br8	*Convolvulus arvensis*	Croatia	KJ573589.1
SecY10	F	*Urtica dioica*	Slovenia	JQ977711.1
SecY11	B	*Urtica dioica*	Italy	JQ977708.1
SecY12	A	*Urtica dioica*	Germany	JQ977707.1
SecY12	H18	*Hyalesthes obsoletus*	Croatia	KJ573587.1
SecY12	GVu1	*Vitis vinifera*	Croatia	KJ573582.1
SecY12	L2753	*unidentified weed*	France	HF969333.1
SecY12	L2505	*Lavandula*	France	HF969332.1

Sequence Variant	Strain	Host	Location	Accession Number
SecY12	H158	*Hyalesthes obsoletus*	France	HF969331.1
SecY12	VU7_1	*Vitis vinifera*	Croatia	HQ413168.1
SecY13	PM1	*Solanum tuberosum*	Montenegro	KU588190.1
SecY13	Carv1	*Convolvulus arvensis*	Georgia	KT184886.1
SecY13	Y3	*Vitis vinifera*	Italy	KJ145389.1
SecY13	CrHo12_601	*Hyalestes obsoletus*	Austria	KJ469715.1
SecY13	Rqg50	*Reptalus quinquecostatus*	Serbia	KC703047.1
SecY13	Vv21	*Vitis vinifera*	Serbia	KC703040.1
SecY13	Rqg60	*Reptalus quinquecostatus*	Serbia	KC703039.1
SecY13	Rpg39	*Reptalus panzeri*	Serbia	KC703037.1
SecY13	H155	*Hyalesthes obsoletus*	France	FN813287.1
SecY13	L1882	*Lavandula angustifolia*	France	FN813286.1
SecY13	GR138	*Vitis vinifera*	Greece	FN813283.1
SecY13	LOZA2	*Vitis vinifera*	Serbia and Montenegro	FN813282.1
SecY14	Amla77	*Vitis vinifera*	Georgia	KT184887.1
SecY15	Y4	*Vitis vinifera*	Italy	KJ145399.1
SecY15	CrHo12_650	*Hyalestes obsoletus*	Austria	KJ469711.1
SecY15	GR13	*Vitis vinifera*	Greece	FN813284.1
SecY16	115	*Vitis vinifera*	Italy	KJ145365.1
SecY16	Vv12_Kn6	*Vitis vinifera*	Austria	KJ469714.1
SecY16	Vv17	*Vitis vinifera*	Serbia	KC703046.1
SecY16	Rqg31	*Reptalus quinquecostatus*	Serbia	KC703045.1
SecY16	LB-L7-3	*Vitis vinifera*	Lebanon	FN813274.1
SecY16	SPF46-09T	*Nicotiana tabacum*	France	FN813273.1
SecY16	BG4560	*Vitis vinifera*	Bulgaria	FN813271.1
SecY17	P53	*Vitis vinifera*	Italy	KJ145364.1
SecY18	136	*Vitis vinifera*	Italy	KJ145362.1
SecY19	Ho13_838	*Hyalestes obsoletus*	Austria	KJ469713.1
SecY19	SB5	*Vitis vinifera*	Croatia	FN813272.1
SecY20	Rqg42	*Reptalus quinquecostatus*	Serbia	KC703044.1
SecY21	GR328	*Capsicum annum*	Greece	FN813285.1
SecY22	L641	*Lavandula angustifolia*	France	FN813281.1
SecY23	L973	*Lavandula sp.*	France	FN813280.1
SecY24	L1884	*Lavandula angustifolia*	France	FN813279.1
SecY25	L646	*Lavandula angustifolia*	France	FN813278.1
SecY26	19-25	*Vitis vinifera*	Germany	AM992094.1
SecY27	23DB_9	*Vitis vinifera*	Croatia	HQ413171.1
SecY28	VU7_2	*Vitis vinifera*	Croatia	HQ413169.1
SecY29	SB1_2	*Vitis vinifera*	Croatia	HQ413166.1
SecY30	21OS_4	*Vitis vinifera*	Croatia	HQ413163.1
SecY31	21OS_2	*Vitis vinifera*	Croatia	HQ413162.1
SecY32	11VZ_3	*Vitis vinifera*	Croatia	HQ413159.1
SecY33	San16_2018	*Vitis vinifera*	Italy	MN557211

**Sequence Variant**	**Strain**	**Host**	**Location**	**Accession Number**
SecY33	Rq_2018	*Reptalus quinquecostatus*	Italy	MN557211
SecY33	W52	*Sonchus sp.*	Italy	MN557211
SecY33	W59	*Convolvulus arvensis*	Italy	MN557211
SecY33	W39	*Picris sp.*	Italy	MN557211
SecY33	W36	*Clematis vitalba*	Italy	MN557211
SecY33	W23	*Centaurea minus*	Italy	MN557211
SecY33	W31	*Matricaria chamomilla*	Italy	MN557211

Table S3. Sequence variants Dataset of the *stamp/secY*-types among '*Ca*. P. solani' strains available in GenBank

Sequnce Variant		Strain	Host	Location
St1	SecY13	Rqg50	*Reptalus quinquecostatus*	Serbia
St1	SecY13	CrHo12_721	*Hyalesthes obsoletus*	Austria
St1	SecY13	PM1	*Solanum tuberosum*	Montenegro
St2	SecY9	Br8	*Convolvulus arvensis*	Croatia
St2	SecY16	Rqg31	*Reptalus quinquecostatus*	Serbia
St2	SecY16	Vv12_Kn6	*Vitis vinifera*	Austria
St2	SecY16	Vv17	*Vitis vinifera*	Serbia
St3	SecY1	P7	*Catharanthus roseus*	Lebanon
St4	SecY13	Rpg39	*Reptalus panzeri*	Serbia
St4	SecY13	Rqg60	*Reptalus quinquecostatus*	Serbia
St4	SecY13	Vv21	*Vitis vinifera*	Serbia
St4	SecY21	GR328	*Capsicum annuum*	Greece
St5	SecY9	GGY	*Vitis vinifera*	Germany
St8	SecY19	Ho13_838	*Hyalesthes obsoletus*	Austria
St8	SecY19	SB5	*Vitis vinifera*	Croatia
St10	SecY1	PO	*Hyalesthes obsoletus*	France
St10	SecY9	LG	*Solanum lycopersicum*	France
St10	SecY33	San16_2018	*Vitis vinifera*	Italy
St10	SecY33	W52	Sonchus sp.	Italy
St10	SecY33	W59	*Convolvulus arvensis*	Italy
St10	SecY33	W36	*Clematis vitalba*	Italy
St10	SecY33	W23	*Centauria minus*	Italy
St10	SecY33	W31	*Matricaria chamomilla*	Italy
St11	SecY12	GVu1	*Vitis vinifera*	Croatia
St11	SecY12	H18	*Hyalesthes obsoletus*	Croatia
St11	SecY15	CrHo12_650	*Hyalesthes obsoletus*	Austria
St11	SecY26	19-25	*Vitis vinifera*	Germany
St12	SecY25	L646	*Lavandula angustifolia*	France
St13	SecY15	GR13	*Vitis vinifera*	Greece
St16	SecY23	L973	*Lavandula angustifolia*	France
St31	SecY16	BG4560	*Vitis vinifera*	Bulgaria
St31	SecY20	Rqg42	*Reptalus quinquecostatus*	Serbia
St35	SecY13	Carv1	*Convolvulus arvensis*	Georgia
St39	SecY14	Amla77	*Vitis vinifera*	Georgia
St59	SecY33	W37_2018	*Convolvulus arvensis*	Italy
St59	SecY33	W39_2018	*Picris hieracioides*	Italy
